# Construction of genotyping-by-sequencing based high-density genetic maps and QTL mapping for fusarium wilt resistance in pigeonpea

**DOI:** 10.1038/s41598-017-01537-2

**Published:** 2017-05-15

**Authors:** Rachit K. Saxena, Vikas K. Singh, Sandip M. Kale, Revathi Tathineni, Swathi Parupalli, Vinay Kumar, Vanika Garg, Roma R. Das, Mamta Sharma, K. N. Yamini, S. Muniswamy, Anuradha Ghanta, Abhishek Rathore, C. V. Sameer Kumar, K. B. Saxena, P. B. Kavi Kishor, Rajeev K. Varshney

**Affiliations:** 1 International Crops Research Institute for the Semi-Arid Tropics, Patancheru, 502 324 India; 2 Professor Jayashankar Telangana State Agricultural University, Rajendranagar, Hyderabad, 500 030 India; 3 Agricultural Research Station (ARS)-Gulbarga, University of Agricultural Sciences (UAS), Raichur, 585 101 India; 4 Osmania University, Hyderabad, 500007 India; 5 School of Plant Biology and Institute of Agriculture, The University of Western Australia, Crawley, WA 6009 Australia

## Abstract

Fusarium wilt (FW) is one of the most important biotic stresses causing yield losses in pigeonpea. Genetic improvement of pigeonpea through genomics-assisted breeding (GAB) is an economically feasible option for the development of high yielding FW resistant genotypes. In this context, two recombinant inbred lines (RILs) (ICPB 2049 × ICPL 99050 designated as PRIL_A and ICPL 20096 × ICPL 332 designated as PRIL_B) and one F_2_ (ICPL 85063 × ICPL 87119) populations were used for the development of high density genetic maps. Genotyping-by-sequencing (GBS) approach was used to identify and genotype SNPs in three mapping populations. As a result, three high density genetic maps with 964, 1101 and 557 SNPs with an average marker distance of 1.16, 0.84 and 2.60 cM were developed in PRIL_A, PRIL_B and F_2_, respectively. Based on the multi-location and multi-year phenotypic data of FW resistance a total of 14 quantitative trait loci (QTLs) including six major QTLs explaining >10% phenotypic variance explained (PVE) were identified. Comparative analysis across the populations has revealed three important QTLs (*qFW11*.*1*, *qFW11*.*2* and *qFW11*.*3*) with upto 56.45% PVE for FW resistance. This is the first report of QTL mapping for FW resistance in pigeonpea and identified genomic region could be utilized in GAB.

## Introduction

Pigeonpea (*Cajanus cajan* (L.) Millsp.) is the sixth most important food legume crop in the world. Pigeonpea cultivars contain about 20 to 23% protein from the total composition of seeds which also possess carbohydrates (65 to 70%) and fiber. Pigeonpea “Dal” is a major source of dietary protein for more than a billion people in the developing world and a rich source of essential amino acids, like methionine, lysine and tryptophan^1–3^. Globally pigeonpea is cultivated on 6.23 million ha (m ha) with an annual production of 4.74 million tons (mt). India is the primary pigeonpea growing country, where more than 85% of the world’s pigeonpea production (3.29 mt, cultivated in 5.06 m ha area) and consumption takes place (http://faostat3.fao.org/home/, as of August 2016). Its productivity measured in experimental plots (~3700 kg/ha) could not be translated in farmers’ fields and average yield (<1000 kg/ha) remained stagnant during the last six decades^3^. This is mainly due to cultivation of pigeonpea with limited or no inputs in heavy diseases pressure by small-holder farmers. Fusarium wilt (FW) and sterility mosaic disease (SMD) are two epidemic diseases in pigeonpea that can cause complete yield losses in susceptible cultivars.

FW caused by *Fusarium udum* Butler has the global importance as it frequently occurs in both India and Africa, the major growing zones in the world. The annual losses due to FW have been reported to be US $ 71 million^4^ and 470, 000 t of grain in India and 30,000 t of grain in Africa^5^. As yield losses due to FW are significant, it is worthwhile to manage and further increase the disease resistance in pigeonpea cultivars. The most pragmatic, economical and environment-friendly approach for FW management is the utilization of genetic resistance^6^. FW resistance in pigeonpea has been found to be controlled by different gene actions while using different donor and suseptible parents. The phenotypic data of segregating mapping populations revealed single to multiple genes with complementary to duplicate gene actions^6, 7^.

Genomics-assisted breeding (GAB) provides strategies to combine indepth genome information with the trait phenotyping data for effective and efficient selection procedures^8^. For implementing GAB, it is essential to develop genetic maps and identify quantitative trait loci (QTLs) or genes associated with desired traits. Subsequently identified QTLs/genes could be deployed in breeding programs through marker-assisted selection (MAS) or some other molecular breeding approach^9–11^. In the case of pigeonpea, limited genetic diversity in cultivated gene pool coupled with inadequate genomic resources caused serious impediments for applying GAB^12^. However, in recent past, ample genomic and genetic resources such as segregating populations (~20 mapping populations, including recombinant inbred lines; introgression lines, multi-parent advanced generation intercross (MAGIC) population and nested association mapping (NAM) population), molecular markers (>3,000, simple sequence repeat (SSR) markers; 15,360 diversity arrays technology (DArT arrays; 10,000 single nucleotide polymorphism (SNP) markers; 1,616 Kompetitive Allele Specific PCR (KASPar) assays; 48 SNPs- based Golden-Gate VeraCode assays and 60K SNPs- based “Axiom®*Cajanus SNP* array”), genetic maps (8 maps with SSRs, DArTs, SNPs), transcriptome assemblies (CcTA v1 and CcTA v2), draft genome sequence etc. have been developed^13, 14^. Recently re-sequencing data through next generation sequencing (NGS) technologies have also been generated for identifying genome-wide variations in parental lines of mapping populations, segregating for economically important traits including FW resistance^15^. Additionally sequencing-based bulked segregant analysis (Seq-BSA) by using a few lines from the mapping population has been used to identify candidate SNPs for resistance to FW and SMD in pigeonpea^16^. In continuation to detect all possible minor and major affect loci contributing to FW resistance in pigeonpea genome, NGS based genetic mapping by employing the entire mapping population has been planned in the present study.

NGS is also providing opportunities to develop high-density genetic maps by deploying high throughput genotyping approaches such as genotyping-by-sequencing (GBS). In fact, GBS has been extensively used in a number of crop species for analysing genetic diversity^17^, developing dense genetic maps^18, 19^, refining the target genomic regions^20^, establishing marker-trait associations^21^ as well as deploying in genomic selection^22, 23^. Therefore, in the present study, GBS based genetic maps for three intra-specific populations have been developed. Populations were phenotyped for one to two years at two to three locations. Subsequently detailed analyses of genotyping data together with phenotyping data have provided consistent genomic regions associated with FW resistance in pigeonpea.

## Results

### Mapping populations and phenotypic evaluation

Three mapping populations including two pigeonpea recombinant inbred lines (PRILs: PRIL_A (ICPB 2049 × ICPL 99050) and PRIL_B (ICPL 20096 × ICPL 332) and one F_2:3_ population (ICPL 85063 × ICPL 87119) along with their parental lines were phenotyped for FW resistance as per the standard procedures. PRIL_A and PRIL_B mapping populations were phenotyped for two years (2012–2013 and 2013–2014) in three replications. However, F_2_ individuals were phenotyped for only one year (2015–2016) at single location.

The phenotyping data of resistant parents (ICPL 20096, ICPB 2049 and ICPL 87119) and susceptible parents (ICPL 332, ICPL 99050 and ICPL 85063) of mapping populations for FW resistance were collected from the previous study^16^. The mean PDI score during 2012–2013 and 2013–2014 among PRIL_A mapping population ranged from 0 to 98.95 at Patancheru location and 5.73 to 92.75 at Gulbarga location (Supplementary Fig. S1(a,b)). The mean PDI score of (2012–2013 and 2013–2014) PRIL_B mapping population showed 0 to 95.6 at Patancheru location, 0 to 68.09 at Gulbarga location and 0 to 94.25 at Tandur location (Supplementary Fig. S1(c–e)). In the case of F_2:3_ population, the PDI score (2014–2015) ranged from 0 to 83.3, whereas the resistant (ICPL 87119) and susceptible parent (ICPL 85063) showed PDI score of 3.36 and 91.6 at Patancheru location, respectively (Supplementary Fig. S1(f)). The range of genetic variations observed in phenotyping data indicated several genes involved in FW resistance and the genetic material is sufficient for QTL mapping (Table 1).

**Table 1:** Phenotypic variation of the FW resistance in pigeonpea recombinant inbred lines (PRILs) and F_2:3_ families.

Mapping population	Location	Year	Mapping population			
			Min (PDI)	Max (PDI)	Average (PDI)	Standard deviation
ICPB 2049 × ICPL 99050 (PRIL_A)	Patancheru	2012–2013	0	97.9	36.8	29.1
	Patancheru	2013–2014	0	100.00	58.80	34.06
	Gulbarga	2012–2013	0	85.50	41.7	22.05
	Gulbarga	2013–2014	11.47	100.00	74.34	20.88
ICPL 20096 × ICPL 332 (PRIL_B)	Patancheru	2012–2013	0	96.80	12.09	19.6
	Patancheru	2013–2014	0	94.40	15.8	20.6
	Gulbarga	2012–2013	0	62.50	13.07	13.88
	Gulbarga	2013–2014	0	73.68	24.09	14.56
	Tandur	2012–2013	0	96	28.14	16.59
	Tandur	2013–2014	0	92.5	50.29	22.1
ICPL 85063 × ICPL 87119 (F_2_)	Patancheru	2015–2016	0	83.3	4.9	40.7

PDI: Percent Disease Incidence score after 90 days of sowing.

### High throughput sequencing and SNP discovery

A total of 46.90 Gb (464.40 million reads), 34.37 Gb (340.30 million reads) and 32.96 Gb (326.34 million reads) clean GBS reads were generated using HiSeq2500 platform for PRIL_A, PRIL_B (sequence data generated, SNPs identified in PRIL_B have been taken from the companion paper being published in Scientific Reports) and F_2_ mapping populations, respectively. The reads from individual progenies ranged from 0.80 to 8.79 million reads in PRIL_A (Supplementary Fig. S2), 0.80 to 5.10 million in PRIL_B, (Supplementary Fig. S3) and 0.80 to 5.46 millions reads for F_2_s (Supplementary Fig. S4). Also, a total of 3.17 (ICPB 2049) and 6.62 (ICPL 99050) million reads of PRIL_A parents, 1.21 (ICPL 20096) and 1.52 (ICPL 332) million reads of PRIL_B parents and 4.12 (ICPL 85063) and 3.61 (ICPL 87119) million reads of F_2_ parents were generated. To identify the genome-wide SNPs, TASSEL-GBS pipeline was used with sequence data for all parents along with respective mapping populations. As a result, a total of 0.10, 0.21 and 0.08 million SNPs were identified in PRIL_A, PRIL_B and F_2_s, respectively (Table 2). Detected SNPs were non-uniformly distributed across different pseudomolecules/CcLGs. For instance, 2,918 (CcLG05) to 21,711 (CcLG11) SNPs in PRIL_A, 5,682 (CcLG05) to 41,292 (CcLG11) SNPs in PRIL_B and 2,580 (CcLG05) to 18,522 (CcLG11) SNPs in F_2_s were distributed across the linkage groups. Further detected SNPs were filtered based on percent heterozygosity, minor allele frequency and missing data information. As a result, a total of 985, 1,789 and 4,209 SNPs were retained for further analysis in PRIL_A, PRIL_B and F_2_s respectively. The numbers of SNPs reduced from several millions to few hundred, due to stringent selection criteria used in the present analysis.

**Table 2:** Distribution of SNP markers on the genetic maps derived from ICPB 2049 × ICPL 99050 (PRIL_A), ICPL 20096 × ICPL 332 (PRIL_B) and ICPL 85063 × ICPL 87119 (F_2_) populations.

Linkage group	ICPB 2049 × ICPL 99050 (PRIL_A)						ICPL 20096 × ICPL 332 (PRIL_B)						ICPL 85063 × ICPL 87119 (F_2_)					
	SNPs identified	Filtered SNPs	SNPs mapped	SNPs mapped (%)	Map length (cM)	Average marker interval (cM)	SNPs identified	Filtered SNPs	SNPs mapped	SNPs mapped (%)	Map length (cM)	Average marker interval (cM)	SNPs identified	Filtered SNPs	SNPs mapped	SNPs mapped (%)	Map length (cM)	Average marker interval (cM)
CcLG01	7366	101	100	99.01	104.15	1.04	14131	75	24	32.00	74.07	3.09	6306	330	42	12.73	193.53	4.61
CcLG02	14805	148	143	96.62	182.54	1.28	32962	219	139	63.47	114.43	0.82	12274	485	41	8.45	228.20	5.57
CcLG03	11345	106	105	99.06	89.35	0.85	27060	172	123	71.51	97.52	0.79	9306	356	7	1.97	78.48	11.21
CcLG04	5294	71	71	100.00	80.63	1.14	11683	87	52	59.77	77.68	1.49	4619	211	22	10.43	78.84	3.58
CcLG05	2918	20	20	100.00	57.83	2.89	5682	26	10	38.46	30.25	3.03	2580	117	9	7.69	95.78	10.64
CcLG06	9142	110	109	99.09	73.83	0.68	21207	205	119	58.05	111.04	0.93	7684	388	47	12.11	102.95	2.19
CcLG07	7949	76	76	100.00	63.56	0.84	16679	148	69	46.62	97.63	1.42	6558	338	16	4.73	119.85	7.49
CcLG08	7391	49	47	95.92	129.27	2.75	16557	174	94	54.02	74.64	0.79	6073	354	21	5.93	121.05	5.76
CcLG09	3985	58	57	98.28	102.89	1.81	8086	74	22	29.73	63.59	2.89	3478	216	18	8.33	99.15	5.51
CcLG10	10176	59	59	100.00	120.79	2.05	17125	182	67	36.81	61.90	0.92	8554	502	20	3.98	115.99	5.80
CcLG11	21711	187	177	94.65	115.72	0.65	41292	427	382	89.46	118.39	0.31	18522	912	314	34.43	212.62	0.68
Average	9280.18	89.55	87.64	98.42	101.87	1.45	19314.91	162.64	100.09	52.72	83.74	1.50	7814	382.64	50.64	10.07	131.49	5.73
Total	102082	985	964	97.87	1120.56	1.16	212464	1789	1101	61.54	921.20	0.84	85954	4209	557	13.23	1446.50	2.60

### SNPs- based genetic maps

**Figure 1:**
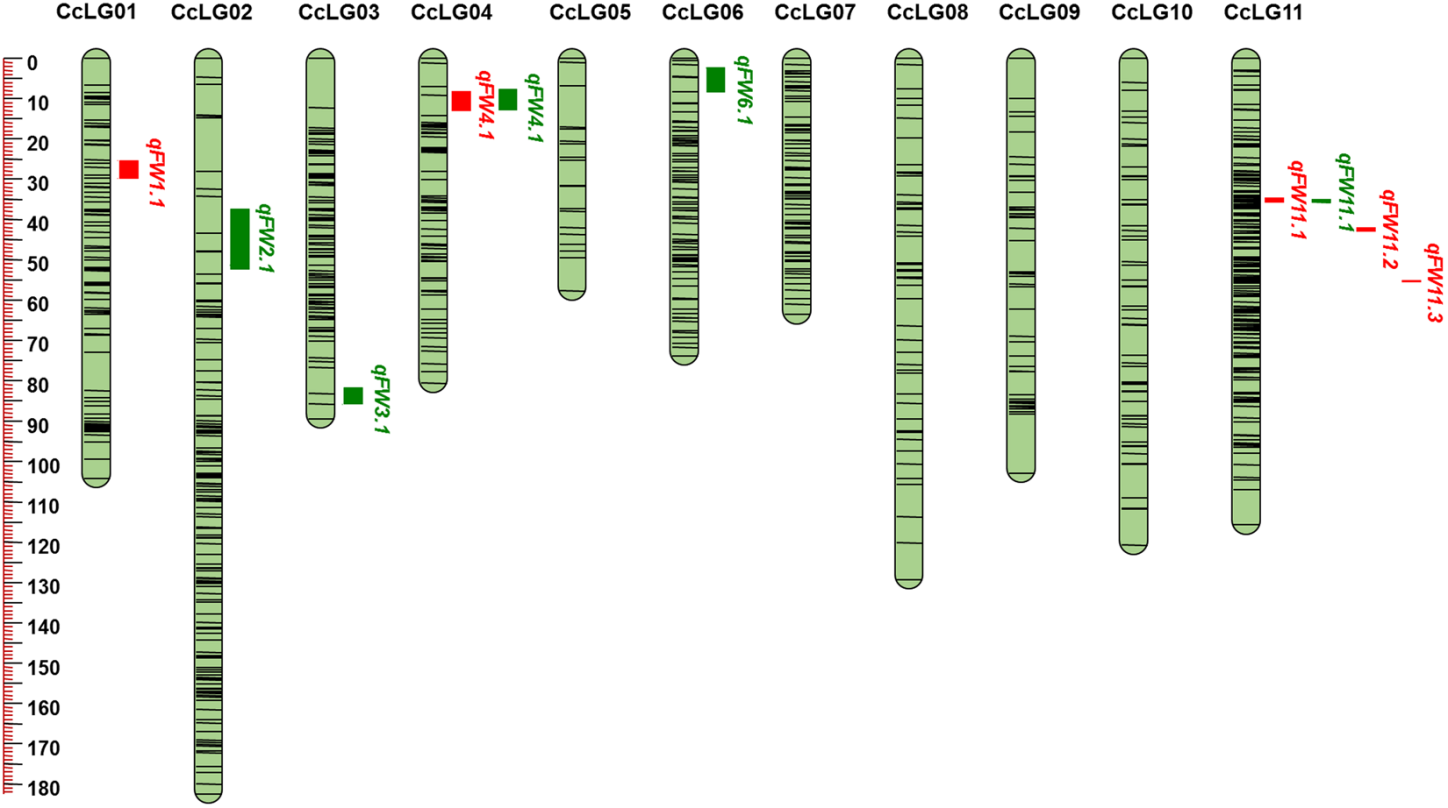
SNPs- based genetic map and distribution of QTLs associated with FW resistance for PRIL_A (ICPB 2049 × ICPL 99050) population. The scale is on the left indicating genetic distance (centi Morgan; cM as unit). The black lines in the linkage groups represent the genetic position of the markers. A total of six linkage groups, namely CcLG01, CcLG02, CcLG03, CcLG04, CcLG06 and CcLG11 possess eight QTLs for FW resistance. QTLs for FW resistance identified at Patancheru and Gulbarga locations were represented as a vertical bar in green and red colors, respectively.

**Figure 2:**
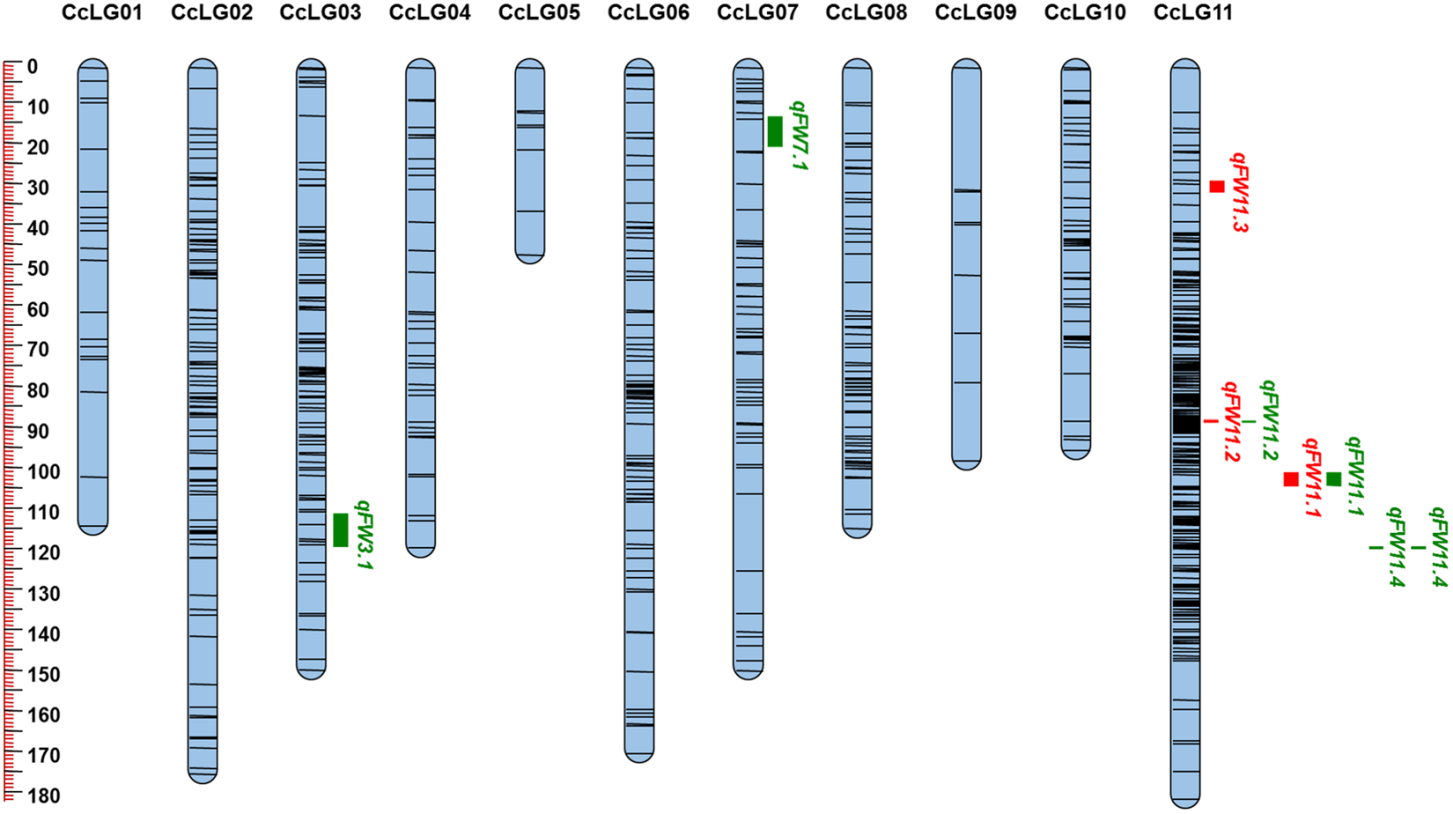
SNPs- based genetic map and distribution of QTLs associated with FW resistance in PRIL_B (ICPL 20096 × ICPL 332). The scale is on the left indicating genetic distance (centi Morgan; cM as unit). The black lines in the linkage groups represent the genetic position of the markers. A total of three linkage groups, namely CcLG03, CcLG07, and CcLG11 possess six QTLs for FW resistance. QTLs for FW resistance identified at Patancheru and Gulbarga locations were represented as a vertical bar in green and red colors, respectively.

**Figure 3:**
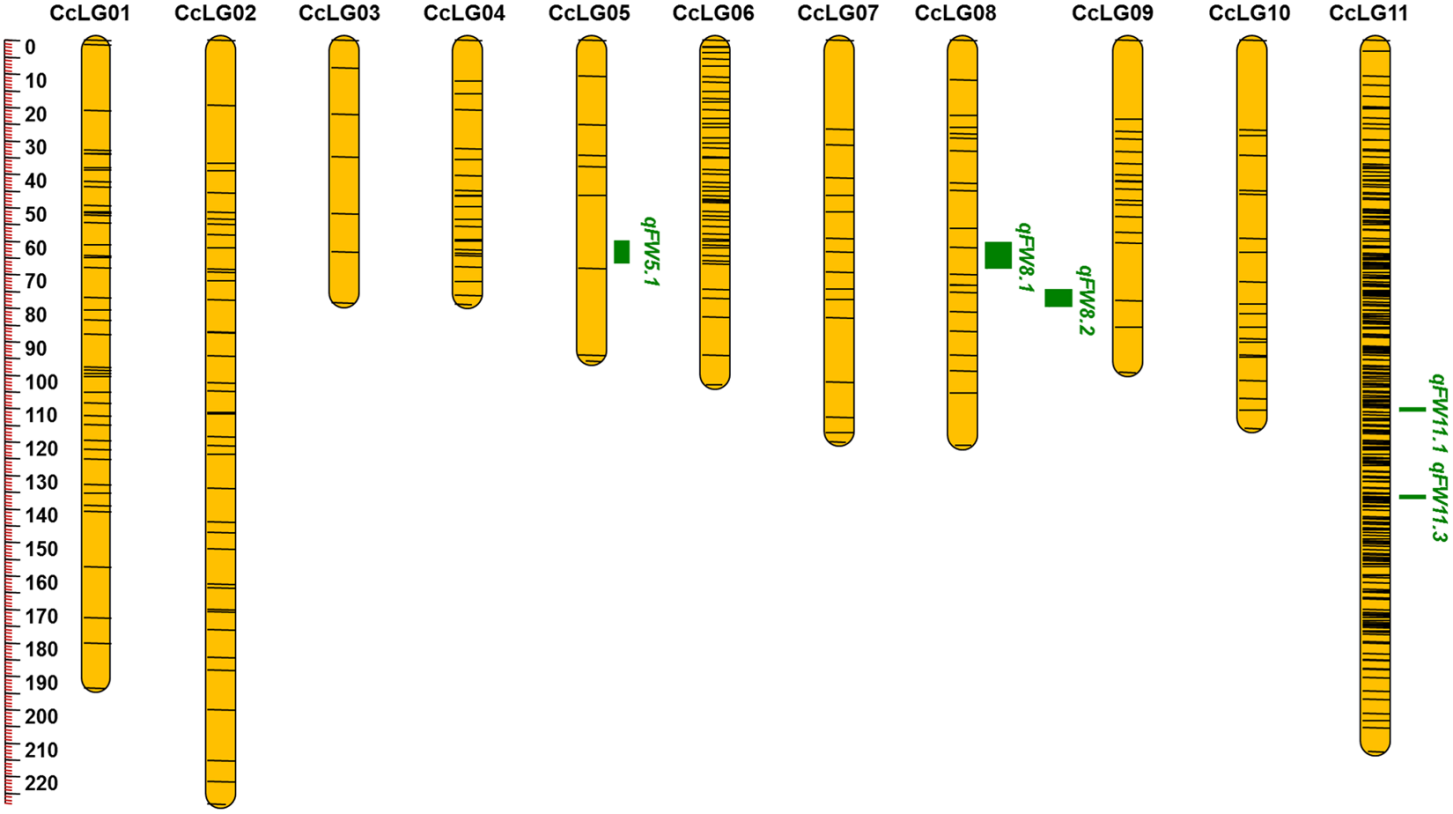
SNPs- based genetic linkage map and distribution of QTLs associated with FW resistance in F_2_ mapping population (ICPL 85063 × ICPL 87119). The scale is on the left indicating genetic distance (centi Morgan; cM as unit). The black lines in the linkage groups represent the genetic position of the markers. Three linkage groups namely, CcLG05, CcLG08 and CcLG11 possess five QTLs for FW resistance. QTLs for FW resistance were represented as a vertical bar in green color.

The identified genome-wide SNPs were evaluated against expected Mendelian segregation ratios through Chi-square analyses in PRIL_A, PRIL_B and F_2_ mapping populations, respectively. The markers with distorted segregation ratio were removed using specific criteria for PRIL_A, PRIL_B and F_2_ (see material and methods for details). As a result, 964, 1101 and 557 SNPs were used for the construction of genetic maps in PRIL_A, PRIL_B and F_2_, respectively. The genetic map derived from PRIL_A was comprised of 964 SNPs distributed on 11 linkage groups (Fig. 1 and Table 2). The PRIL_A genetic map encompassed 1120.56 cM, with linkage groups ranged from 57.83 cM (CcLG05) to 182.54 cM (CcLG02). The number of SNPs mapped to each linkage group varied from 20 SNPs in CcLG05 to 177 SNPs in CcLG11, with a mean of 88 SNPs per linkage group. The inter-marker distance in linkage group ranged from 0.65 (CcLG11) to 2.89 (CcLG05) with a mean inter-marker distance of 1.16. The genetic map for PRIL_B comprised of 1101 SNPs over 11 linkage groups with total map length of 921.20 cM (Fig. 2 and Table 2). The length of individual linkage group was ranged from 30.25 cM (CcLG05) to 118.39 cM (CcLG11). The number of SNPs mapped to each linkage group varied from 10 SNPs in CcLG05 to 382 SNPs in CcLG11, with a mean of 100 SNPs per linkage group. The inter-marker distance in linkage groups ranged from 0.31 (CcLG11) to 3.09 (CcLG01) with a mean inter-marker distance of 0.84. The genetic map in F_2_ population consisted of 557 SNPs with a total length of 1446.50 cM (Fig. 3 and Table 2 **)**. The number of SNPs in each linkage group ranged from 7 (CcLG03) to 314 (CcLG11) with the map length of 78.48 cM (CcLG03) to 228.20 cM (CcLG02). The average marker distance in linkage groups ranged from 0.68 cM (CcLG11) to 11.20 cM (CcLG03) with mean average inter-marker distance of 2.60 cM, across linkage groups. The developed linkage maps in all three mapping populations together with respective phenotyping data were used for QTL analysis.

### QTLs for FW resistance

Phenotyping data together with SNP genotyping data were used for QTL analysis in PRIL_A, PRIL_B and F_2_ populations using composite interval mapping (CIM). Based on the phenotypic variance explained (PVE), identified QTLs were classified as major (≥10% PVE) and minor QTLs (<10% PVE). The identifed QTLs were also classified as stable (appeared in more than one location) and consistent QTLs (appeared in more than one year). For each population details on QTLs identified have been explained below.

### QTLs in PRIL_A

A total of eight QTLs were identified in PRIL_A dispersed on six linkage groups (CcLG01, CcLG02, CcLG03, CcLG04, CcLG06, and CcLG11) with PVE ranged from 6.55 (*qFW1*.*1*) to 14.67% (*qFW3*.*1*) (Table 3). Four QTLs, namely *qFW3*.*1* (14.67%), *qFW6*.*1* (10.71%), *qFW11*.*1* (12.11%) and *qFW11*.*2* (10.04%) were identified as major effect QTLs and remaining four QTLs showed minor effects (*qFW1*.*1*, *qFW2*.*1*, *qFW4*.*1* and *qFW11*.*3*) for FW resistance. Two QTLs were also identified as stable QTLs (appeared in more than one location), namely, *qFW4*.*1* (Patancheru 2012–2013 and Gulbarga 2013–2014) and *qFW11*.*1* (Gulbarga 2012–2013 and Patancheru 2013–2014). However, none of identified QTLs was found consistent QTL across the years and at both the locations.

**Table 3:** Summary on QTL mapping for FW resistance on the genetic map derived from PRIL_A (ICPB 2049 × ICPL 99050).

QTL	Location	Year	Linkage group	Position (cM)	Marker interval	QTL size (cM)	PVE%	Additive effect	LOD value
*qFW1*.*1*	Gulbarga	2013–2014	CcLG01	26.01	S1_2827280–S1_4263752	4.5	6.55	0.07	2.50
*qFW2*.*1*	Patancheru	2013–2014	CcLG02	43.41	S2_16115010–S2_15580586	15.1	7.58	−0.08	2.95
*qFW3*.*1*	Patancheru	2012–2013	CcLG03	84.21	S3_18695411–S3_17153283	4.2	14.67	−0.11	3.05
*qFW4*.*1*	Patancheru	2012–2013	CcLG04	9.21	S4_597553–S4_1108184	5.2	7.45	−0.08	3.04
	Gulbarga	2013–2014	CcLG04	9.21	S4_597553–S4_1108184	4.7	7.40	−0.07	2.98
*qFW6*.*1*	Patancheru	2013–2014	CcLG06	5.71	S6_22726005–S6_23553522	6.1	10.71	−0.10	2.81
*qFW11*.*1*	Gulbarga	2012–2013	CcLG11	35.01	S11_37262913–S11_37133265	1.1	12.11	0.08	2.97
	Patancheru	2013–2014	CcLG11	35.61	S11_43777543–S11_37133265	0.7	8.19	0.09	2.94
*qFW11*.*2*	Gulbarga	2012–2013	CcLG11	42.81	S11_20607023–S11_16809228	0.9	10.04	0.08	3.25
*qFW11*.*3*	Gulbarga	2013–2014	CcLG11	55.41	S11_4243778–S11_22408748	0.4	8.17	0.08	2.78

### QTLs in PRIL_B

QTL mapping analysis in PRIL_B identified a total of six QTLs on three linkage groups (CcLG03, CcLG07, and CcLG11) and PVE ranged from 7.92 (*qFW3*.*2*) to 15.26% (*qFW7*.*1*) (Table 4). Out of six, two QTLs, namely *qFW7*.*1* (15.26%) and *qFW11*.*4* (14.72%) were identified as major QTLs. Whereas, four QTLs (*qFW3*.*2*, *qFW11*.*3*, *qFW11*.*2* and *qFW11*.*1*) with PVE ranged from 7.92 to 9.71% were defined as minor effect QTLs. A single consistent (appeared in more than one year/season) QTL (*qFW11*.*4* appeared at Patancheru 2012–2013 and 2013–2014) and two stable QTLs namely *qFW11*.*1* (Gulbarga 2012–2013 and Patancheru 2012–2013) and *qFW11*.*2* (Gulbarga 2012–2013 and Patancheru 2012–2013) were detected in PRIL_B.

**Table 4:** Summary on QTL mapping for FW resistance on the genetic map derived from PRIL_B (ICPL 20096 × ICPL 332).

QTL	Location	Year	Linkage group	Position (cM)	Marker interval	QTL size (cM)	PVE%	Additive effect	LOD value
*qFW3*.*2*	Patancheru	2012–2013	CcLG03	73.91	S3_7852159–S3_7852138	5.2	7.92	−0.15	2.91
*qFW7*.*1*	Patancheru	2013–2014	CcLG07	10.31	S7_6202998–S7_4645510	4.9	15.26	−0.14	3.00
*qFW11*.*1*	Gulbarga	2012–2013	CcLG11	65.91	S11_23244327–S11_45340470	2.2	9.16	0.13	2.99
	Patancheru	2012–2013	CcLG11	65.91	S11_23244327–S11_45340470	2.1	8.63	0.12	2.91
*qFW11*.*2*	Gulbarga	2012–2013	CcLG11	57.21	S11_8867457–S11_20235547	0.3	8.94	−0.13	2.66
	Patancheru	2013–2014	CcLG11	57.21	S11_8867457–S11_20235547	0.3	9.71	−0.12	2.94
*qFW11*.*3*	Gulbarga	2013–2014	CcLG11	18.81	S11_5757399–S11_2019429	1.9	8.89	0.09	2.84
*qFW11*.*4*	Patancheru	2012–2013	CcLG11	77.71	S11_7119684–S11_10698013	0.3	9.38	−0.15	2.86
	Patancheru	2013–2014	CcLG11	77.71	S11_7119684–S11_10698013	0.3	14.72	−0.17	4.70

### QTLs in F_2_ population

In the case of F_2_ population, a total of five QTLs, one on CcLG05 (*qFW5*.*1*), two on CcLG08 (*qFW8*.*1* and *qFW8*.*2*) and two on CcLG11 (*qFW11*.*1* and *qFW11*.*3*) were identified (Table 5). The PVE by the QTLs ranged from 2.75 (*qFW8*.*1*) to 56.45% (*qFW11*.*3*). Based on the PVE classification two major QTLs namely *qFW5*.*1* (15.68%) and *qFW11*.*3* (56.45%) were reported in the F_2_ population.

**Table 5:** Summary on QTL mapping for FW resistance on the genetic map derived from F_2_ (ICPL 85063 × ICPL 87119) population.

QTL	Location	Year	Linkage group	Position (cM)	Marker interval	QTL size (cM)	PVE%	Additive effect	Dominance effect	LOD value
*qFW5*.*1*	Patancheru	2015–2016	CcLG05	62.31	S5_3597126–S5_3598272	2.1	15.68	26.20	−26.77	24.52
*qFW8*.*1*	Patancheru	2015–2016	CcLG08	62.81	S8_6388803–S8_7664779	7.7	2.75	−2.33	−2.52	3.46
*qFW8*.*2*	Patancheru	2015–2016	CcLG08	75.31	S8_17995219–S8_3841197	5.1	4.26	−2.53	−2.15	4.06
*qFW11*.*1*	Patancheru	2015–2016	CcLG11	124.61	S11_41835381–S11_33516474	0.8	8.85	1.68	−3.07	2.65
*qFW11*.*3*	Patancheru	2015–2016	CcLG11	110.61	S11_12662418–S11_21940836	1.1	56.45	25.94	−26.85	22.11

### Candidate genomic regions for FW resistance

**Figure 4:**
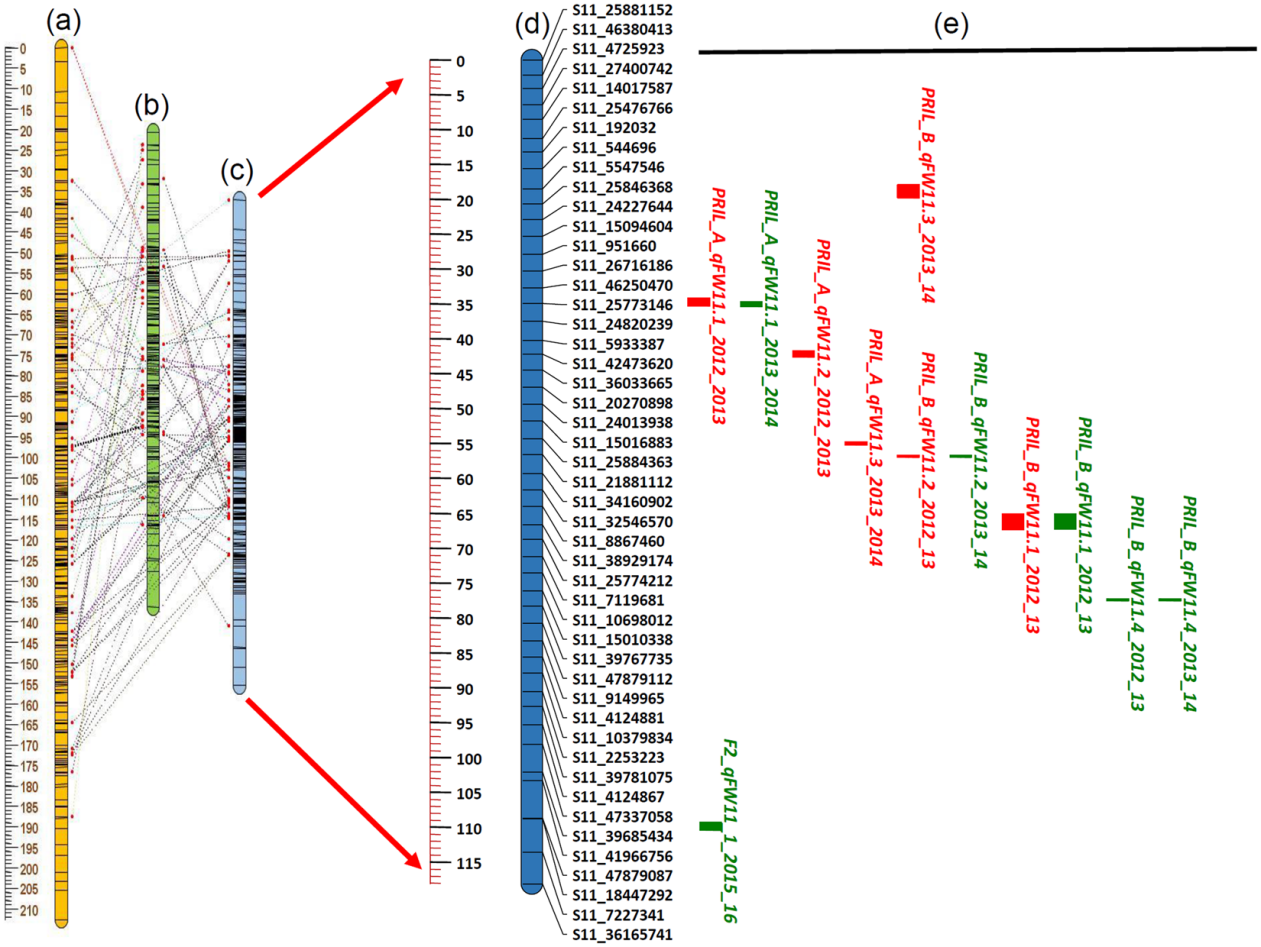
Iterative map of CcLG11 and distribution of QTLs associated with FW resistance among three mapping populations. The Iterative map of CcLG11 was created using Biomercator version 4.2 using the genetic information of all three linkage maps (**a**) PRIl_A, (**b**) PRIL_B, and (**c**) F_2_. Based on the presence of common markers (connected with dotted arrow) among three populations a genetic linkage map (**d**) of ~118 cM was developed. Marker names are indicated to the right of the linkage groups, and the map distances in cM are shown to the left of the linkage groups. (**e**) A total of 13 QTLs identified for FW resistance at Patancheru and Gulbarga locations were represented as a vertical bar in green and red colors, respectively. A QTL is named as *qFWY*.*a*-YYYY_YY, with ‘FW’ being the trait abbreviation fusarium wilt, ‘Y’ the number of the linkage group, ‘a’ the letter to specify different QTLs for the same trait in one linkage group (CcLG), and ‘YYYY_YY’ the year in which the trait was phenotyped. To represent the QTLs of different experiments, PRIL_A, PRIL_B and F_2_ were added as prefix before the name of the QTLs.

Composite Interval Mapping (CIM) analysis in the above mentioned populations have provided a total of 14 significant QTLs across all the linkage groups except CcLG09 and CcLG10 (Supplementary Table S1). Out of 14 QTLs, 7 QTLs showed major effects and the proportion of PVE by individual QTLs ranged from 10.04 (*qFW11*.*4*) to 56.45%. (*qFW11*.*1*). Comparative analysis of QTLs identified between PRIL_A and PRIL_B revealed three QTLs on CcLG11 (*qFW11*.*1*, *qFW11*.*2* and *qFW11*.*3*) as common in both mapping populations based on the SNP positions in genome assembly. Across all the three populations i.e. PRIL_A, PRIL_B and F_2_, only one QTL, namely *qFW11*.*1* was found common. To identify the common genomic regions on CcLG11 an iterative map was created using the genetic information of all three genetic maps (for PRIL_A, PRIL_B and F_2_ population). Based on the presence of common markers among three populations on CcLG11, an iterative linkage map of ~118 cM length has been created. As a result, three QTLs from PRIL_A, four QTLs from PRIL_B and two QTLs from F_2_ mapping population were visualized on CcLG11. Comparative analysis across all three mapping populations resulted in the identification of three important QTLs namely *qFW11*.*1*, *qFW11*.*2* and *qFW11*.*3* (Fig. 4). These genomic regions on CcLG11 can be considered as first choice for the breeder to introgress FW resistance in susceptible cultivars through GAB.

## Discussion

To understand the genetic nature of FW resistance in pigeonpea few studies have been conducted in past^6, 7^. Efforts in identifying trait associated markers through BSA or through marker association analysis have also been published. For instance, two random amplified polymorphic DNA (RAPD) markers^24^, four sequence characterized amplified region (SCAR) markers^25^ and six simple sequence repeat (SSR) markers^26, 27^ were reported for FW resistance. Recently, Seq-BSA together with non-synonymous SNP (nsSNPs) based approach was applied to identify four candidate SNPs in four genes associated with FW resistance^16^. However, no QTL mapping experiment has been conducted till date to find out genome-wide regions controlling FW resistance in pigeonpea. The identification and introgression of QTLs for FW resistance in popular but susceptible pigeonpea varieties/parental lines though GAB is a fast-track approach to the development of new breeding lines with enhanced resistance to FW. Therfore, this study aimed at identifying major and stable FW resistant QTLs from different donor background through GBS based genetic maps and multi-year and multi-location phenotyping.

Mapping populations in the present study were phenotyped in one to two crop seasons at one to three locations for FW resistance. Detailed analysis of phenotyping data showed that parents were true to the type in terms of disease reactions and huge variations among progenies were observed across locations and years. The results indicated that sufficient variatons were present to map the FW resistance. However, due to location × environment interations, huge variaion was observed in the average PDI score. The phenotypic variation ultimately, affects the identifcation of stable QTLs across the years.

Construction of dense genetic maps is useful in trait mapping studies, including fine mapping, positional cloning, construction of genome assembly and further improvement of genome sequences^28, 29^. The current availability of more than >3000 SSR markers; diversity arrays technology (DArT) array with 15,360 features; 1616 Kompetitive Allele Specific PCR (KASP) provides an opportunity to develop high-density genetic maps^30^. However, due to the low level of genetic polymorphism in *Cajanus* spp, it was difficult to develop dense genetic maps. In this direction, six SSR-markers based intra-specific genetic maps with 59 to 140 SSR loci were constructed, while the consensus map constructed using six component maps had only 339 SSR markers^31^. To overcome the problems mentioned above at some extent, first high density genetic map with 875 KASP and 35 SSR markers was developed for an inter-specific population with an average inter marker distance of 1.11 cM^28^. Therefore GBS seems to be a promising approach for the construction of dense intra-specific genetic maps in pigeonpea. Construction of GBS based high density genetic maps is the common approach in the majority of the crop plants^30, 32, 33^. In the present study GBS approach was used for the construction of three genetic maps for intra-specific populations.

In the present study, we have used GBS approach to discover and genotype SNPs in three mapping populations, namely PRIL_A, PRIL_B and F_2_s. Sequencing of mapping populations resulted in the identification of 0.10, 0.21 and 0.08 million SNPs in PRIL_A, PRIL_B and F_2:3_, respectively. Detected SNPs in the present analysis were comparatively higher to any of the previously reported genetic mapping studies in pigeonpea^28^. Further, a stringent selection criterion including missing percentage, minor allele frequency and percent heterozygosity was adopted to filter out the SNPs to select the panel of robust SNPs for constructing high-density genetic maps. The adopted stringent criteria reduce the number of SNPs from millions to few hundred, however, this is very common in GBS data, and similar results have been reported in many other GBS based genetic mapping studies^19, 20^. In summary, in the present study, a total of 985 (0.96%), 1789 (0.84%) and 4209 SNPs (4.89%) were finally obtained in PRIL_A, PRIL_B and F_2_ respectively. The number of mapped markers was low in comparison to the identified SNPs between parental lines may be due to limited sequencing depth used for GBS. The numbers of SNPs in F_2_ were found higher in comparison to PRILs and this may be attributed to different parameters used to select SNPs in RIL and F_2_.

As compared to earlier genetic maps, dense genetic maps were constructed using 964 (97.86% SNPs mapped), 1101 (61.54% SNPs mapped) and 557 (13.23% SNPs mapped) SNP loci for PRIL_A, PRIL_B and F_2_ populations, respectively. The number of SNPs mapped in F_2_ population was quite low as compared to RILs and this may be due to segregation distortion of SNPs in F_2_s. Similar observations were also reported in maize while constructing GBS based genetic map for F_2_ population^34^. The current dense genetic maps (964 SNPs in PRIL_A), 1101 SNPs in PRIL_B and 557 SNPs in F_2_) will be useful for the development of high-density consensus map and fine mapping of QTLs^28, 31, 35^. However, in the present study we could not develop a consensus map due to lack of common markers across the mapping populations. This may be because of inherent nature of GBS, which takes random sites in the genome for detecting polymorphism.

The developed genetic maps have shown big gaps (higher marker intervals) on few linkage groups (CcLG05 and CcLG08 in PRIL_A; CcLG01 and CcLG05 in PRIL_B). In the case of F_2_ mapping population, two linkage groups showed >10 cM of inter marker distance (CcLG03 and CcLG05), which was quite high for QTL mapping experiments. In this context, currently developed 60K “Axiom®*Cajanus SNP* array” (unpublished) will be useful in enriching these genetic maps in future for trait discovery programs in pigeonpea.

QTL mapping for FW resistance have revealed 14 significant QTLs with six of these QTLs showed major effects with PVE of more than 10%. The identified QTLs in the present study are novel as this is the first report of mapping QTLs for FW resistance in pigeonpea. As expected, only few QTLs were stable and consistent and this might be due to the presence of environment interactions/pathogenic variability across the locations. It is interesting to note that we found three QTLs on CcLG11 (*qFW11*.*1*, *qFW11*.*2*, and *qFW11*.*3*) with significant effects while using three different mapping populations. Comparative analysis of the present study with the Seq-BSA based FW resistance gene mapping^16^ revealed two earlier identified SNPs at CcLG11 (32606065 bp and 35228097 bp) in the close vicinity or in the identified QTL region of *qFW11*.*1* (37133265–43777543 bp in PRIL_A; 23244327–45340470 bp in PRIL_B and 33516474–41835381 bp in F_2_). However, other SNPs present on the CcLG02 (27426866 bp and 27861114 bp) identified earlier using seq-BSA approach were comparatively far from the QTL *qFW2*.*1* (15580586–16115010 bp) identified in this study. These comparative analyses across two different studies revealed the importance of the genomic region present at CcLG11 in controlling FW resistance. It was surprising to note that despite using GBS based genotyping approach and multi-location and multi-year phenotypic data sets we had detected QTLs with upto 15.26 PVE in RILs, which was quite low and thus, indicating the complex nature of FW resistance. In the case of the F_2_ population, a QTLs namely, *qFW11*.*3* with 56.45 PVE was detected. However, the same QTLs was detected as minor QTLs in both of the RIL populations. The major reason for such differences in PVE showed in RILs and F_2_ is possibly due to the difference in resolution of the genetic maps, as high density mapping can reduce the false positives in QTL detection^36^. Altogether, these results suggested that the dense genetic map provides accurate detection of QTLs for FW resistance in pigeonpea.

In conclusion, three intra-specific dense genetic maps were constructed using GBS approach. The marker density in the maps was ranged from one in 0.84 to 2.60 cM. Based on these genetic maps and phenotypic data, 14 significant QTLs were identified with PVE ranged from 2.65% to 56.45%. Six of these QTLs showed major effects with PVE of more than 10% each. Comparative analysis revealed three important QTLs namely *qFW11*.*1*, *qFW11*.*2* and *qFW11*.*3* detected across the populations. All the QTLs identified in the present study for FW resistance were novel. Some of these QTLs can be deployed in GAB for development of FW resistant pigeonpea genotypes and for molecular dissection of FW resistance.

## Methods

### Mapping populations

Two recombinant inbred line (RIL) populations generated by crossing ICPB 2049 (FW susceptible) × ICPL 99050 (FW resistant) (designated as PRIL_A), ICPL 20096 (FW resistant) × ICPL 332 (FW susceptible) (designated as PRIL_B) comprising of 188 individuals using single seed descent method^12^. Together with RILs, one early generation (F_2_) mapping population consisting of 168 lines by crossing ICPL 85063 (FW susceptible) × ICPL 87119 (FW resistant) was also utilized in the present study.

### Phenotyping for FW resistance

PRIL_A were phenotyped in sick plot nursery at two locations namely Patancheru (Telangana State, India) and Gulbarga (Karnataka, India) for FW resistance during crop season 2012–2013 and 2013–2014. PRIL_B was phenotyped at three different locations, namely Patancheru (Telangana State, India), Gulbarga (Karnataka, India) and Tandur (Telangana State, India) during crop season 2012–2013 and 2013–2014. Field phenotyping of mapping populations was done in sick plot nurseries with standard procedures^37^. The phenotyping of RILs was conducted in three replications using randomized complete block design (RCBD). The disease score of highly susceptible local checks at regular intervals was recorded. The phenotyping data of parental lines of RIL mapping populations for FW resistance were collected from the previous study^16^. The phenotyping of F_2_ population was completed in F_2:3_ plants to avoid the loss of the plants and to get replicated data during the year 2015–2016 at Patancheru (Telangana State, India). BLUPs were estimated from multi-location data and the arcsine transformed values used for QTL analysis. The experimental plots were four meters long with a row to row spacing of 75 cm and 20 cm spacing between plants. Individual plants in each population were evaluated based on the percent disease incidence (PDI) score at 90 days after sowing (DAS).

### DNA isolation and sequencing of GBS libraries

Two to three young leaves from individual plants in PRIL_A (188 individual), and F_2_s (168 individuals) and parental lines were used to isolate genomic DNA using NucleoSpin Plant II kit (Macherey-Nagel, Düren, Germany). The GBS data of PRIL_B have been taken from the companion paper being published in Scientific Reports). The quality and quantity of DNA was checked on 0.8% agarose gel and then using Qubit 2.0 fluorometer (Thermo Fisher Scientific Inc., USA).

GBS approach was used for simultaneous SNP discovery and genotyping of mapping populations^38^. 10 ng genomic DNA from each sample was restriction digested using *ApeKI* (recognition site: G/CWCG) endonuclease. The digested product was ligated with uniquely barcoded adaptors using T4 DNA ligase enzyme and was further incubated at 22 °C for 1 h and heated at 65 °C for 30 min to inactivate the T4 ligase. Such digested ligated products from each sample were mixed in equal proportion to construct the GBS libraries, which were then amplified, purified to remove excess adapters. The DNA libraries were sequenced on HiSeq 2500 platform (Illumina Inc, San Diego, CA, USA) to generate genome-wide sequence reads.

### SNP genotyping

Sequence reads from raw FASTQ files were used for SNP identification and genotyping using reference based GBS analysis pipeline implemented in TASSEL v4.0^39^. The draft genome sequence information of pigeonpea variety ‘Asha’ was used as reference assembly^40^. Briefly, the sequencing reads were searched for perfectly matched barcode with the expected four base remnant of the enzyme cut site using the in-house script. The barcode containing reads were sorted, de-multiplexed according to barcode sequence and trimmed to first 64 bases starting from enzyme cut site. Further, those reads containing ‘N’ within first 64 bases were rejected. The remaining good quality reads (called as tags) were aligned against the draft genome sequence of pigeonpea using Burrows-Wheeler Alignment tool (BWA)^41^. The alignment file was then processed through GBS analysis pipeline for SNP calling and genotyping.

The individuals with less than 80 Mb data were not selected for further analysis to avoid false positive detection. Due to the presence of the different levels of heterozygosity in PRIL_A and F_2_, different criteria were used to filter SNPs identified in these populations. In the case of PRIL_A, lines with more than 50% missing data and minor allele frequency (MAF) of ≤0.3 were filtered out. In F_2_, SNPs with contrasting alleles in parental genotypes and having <30% missing data were retained for further study. Further, imputation of missing data was carried out using FSFHap algorithm implemented in TASSEL v4.0^39^ in both the mapping populations. The imputed SNPs were further filtered with minor allele frequency (MAF) cut off of 0.2 to remove missing data, and such filtered SNPs were used for genetic mapping and QTL studies.

### Construction of genetic maps

For the linkage analysis, identified SNPs were first tested against the expected segregation ratios. The homozygous SNPs with respect to the two parents were expected to segregate in a 1:2:1 ratio in F_2_ population, whereas a pair of homozygous SNP alleles, in PRIL_A was expected to segregate in a 1:1 ratio. Markers showing significant segregation distortion (P < 0.01, χ^2^ test) were removed from PRIL_A.The genetic map information of PRIL_B have been taken from the companion paper being published in Scientific Reports)﻿. As segregation distortion of SNPs was the major issue in F_2_s, therefore, SNPs showing expected segregation at a *P*-value of <10^−9^ were retained and used for the construction of genetic map^34^.

To construct the genetic maps, Joinmap V4.0 was used. The grouping and ordering of markers were carried out using regression mapping algorithm with a maximum recombination frequency of 0.4 at minimum logarithm of odds (LOD) value of 3 using the command “LOD groupings” and “create groups for mapping” into respective linkage groups (LG). The Kosambi mapping function was used to convert recombination fraction into map units. After developing the framework genetic maps with the marker orders, the unmapped markers were integrated into different linkage groups at recombination frequency up to 50% using ripple command. The visualization of genetic maps was done using the software MapChart 2.30^42^. Iterative map projection of specific linkage group was created using BioMercator V4.2^43^.

### QTL analysis and visualization

QTL analysis was conducted with WinQTLCart2.5 software program (http://statgen.ncsu.edu/qtlcart/WinQTLCart) using composite interval mapping (CIM). The CIM analysis was run using 1.0 cM as scanning interval between markers and tentative QTL with a window size of 10.0, model 6, 1,000 permutations at a whole genome-wide significance level of *P* < 0.05. The location of each QTL was determined according to its LOD peak location and surrounding region. The LOD score values (2.5) were used to determine the significance of QTL. A QTL is named as *qFWY*.*a* with ‘FW’ being the trait abbreviation fusarium wilt, ‘Y’ the number of the linkage group, ‘a’ the letter to specify different QTLs for the same trait in one linkage group based on the physical positions of left and right QTL linked markers the nomenclature of QTL was designated as novel or not. MapChart 2.30^42^ was used to project QTLs on the linkage groups.

## Electronic supplementary material

Supplimentary Informations
