# Supplementary material for: Construction of genotyping-by-sequencing based high-density genetic maps and QTL mapping for fusarium wilt resistance in pigeonpea
Source: Sci Rep. 2017 May 15;7:1911. doi: 10.1038/s41598-017-01537-2 (PMC5432509; doi:10.1038/s41598-017-01537-2)
Supplement: Supplementary file 1 — Supplimentary Informations [file 41598_2017_1537_MOESM1_ESM.doc]

**Supplementary Information**

**Full Paper**

Construction of genotyping-by-sequencing based high-density genetic maps and QTL mapping for fusarium wilt resistance in pigeonpea

**Running Title**

Genetic dissections of fusarium wilt resistance in pigeonpea

Rachit K. Saxena^1,†^, Vikas K. Singh^1,†^ Sandip M. Kale^1^, Revathi Tathineni^1^, Swathi Parupalli^1^, Vinay Kumar^1^, Vanika Garg^1^, Roma Rani Das^1^, Mamta Sharma^1^, K. N. Yamini^2^, S Muniswamy^3^, Anuradha Ghanta^2^, Abhishek Rathore^1^, C. V. Sameer Kumar^1^, K. B. Saxena^1^, P. B. Kavi Kishor ^4^, Rajeev K. Varshney^1, ,5, *^

^1^International Crops Research Institute for the Semi-Arid Tropics, Patancheru - 502 324, India

^2^ Professor Jayashankar Telangana State Agricultural University, Rajendranagar, Hyderabad - 500 030, India

^3^Agricultural Research Station (ARS)-Gulbarga, University of Agricultural Sciences (UAS), Raichur - 585 101, India

^4^Osmania University, Hyderabad 500007, India

^5^School of Plant Biology and Institute of Agriculture, The University of Western Australia, Crawley, WA, 6009 Australia

*To whom correspondence should be addressed. Tel: 91-40-30713305; Fax: 91-40-30713074

Email: [r.k.varshney@cgiar.org](mailto:r.k.varshney@cgiar.org);

†These authors contributed equally to this work


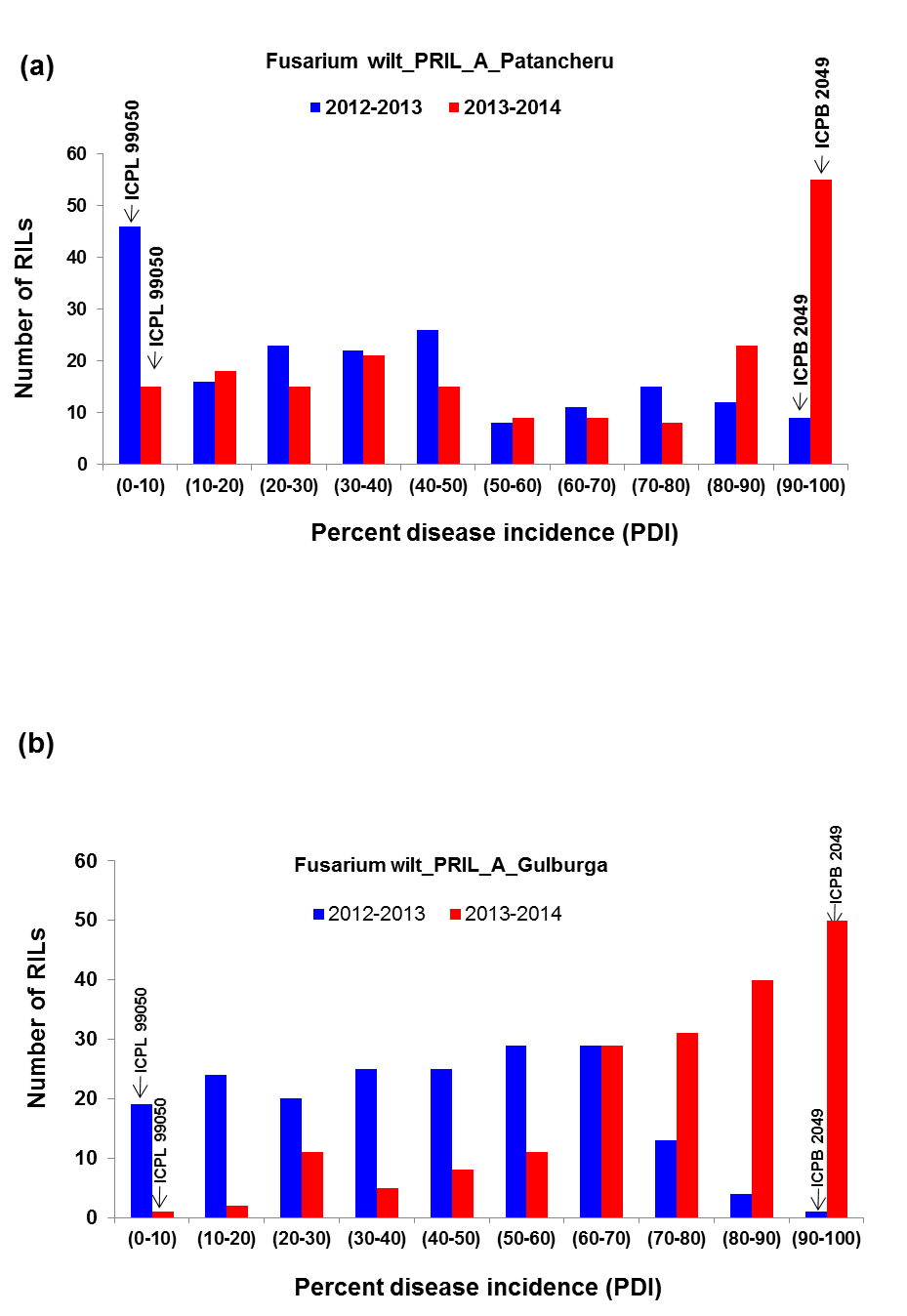


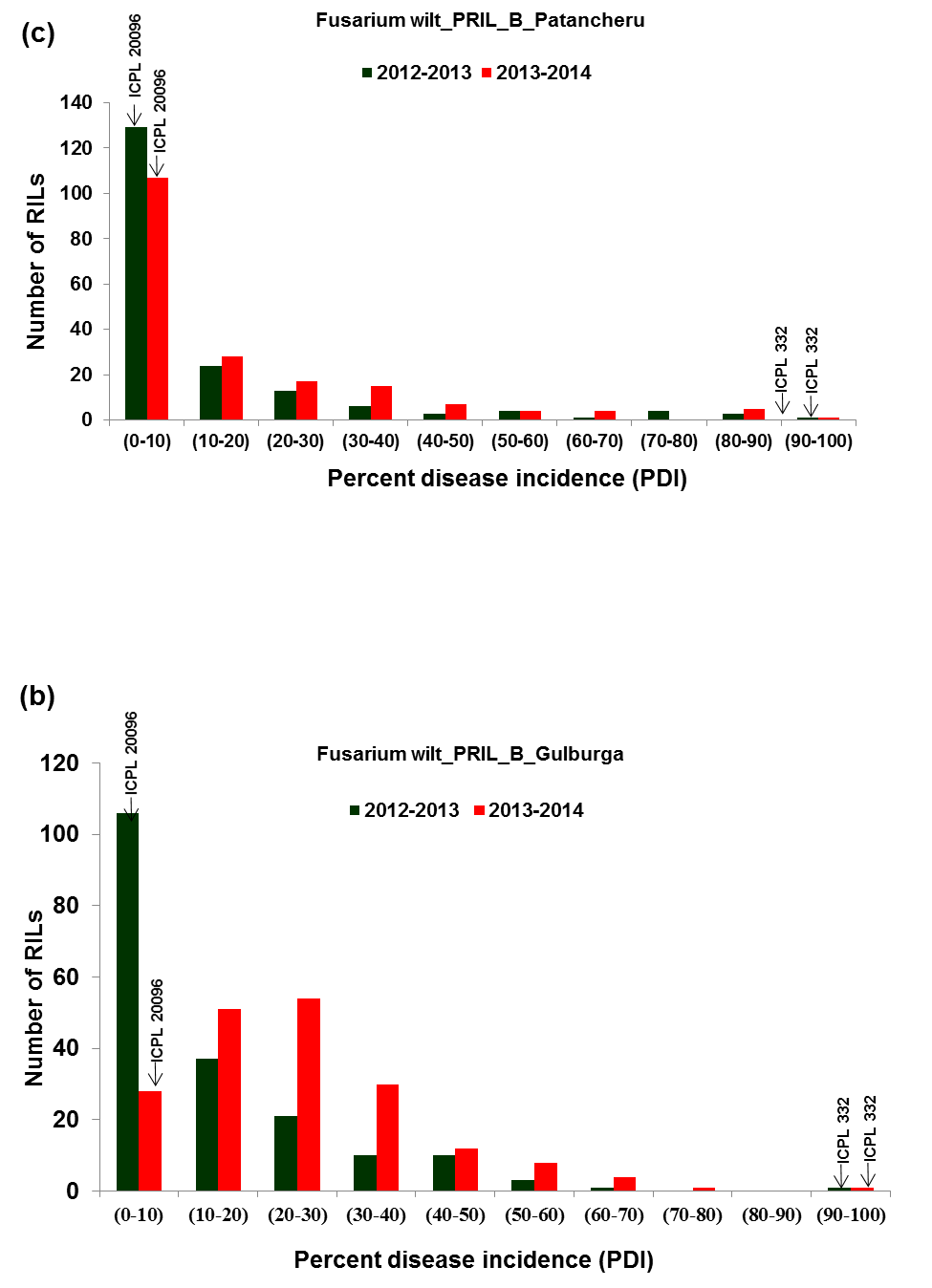


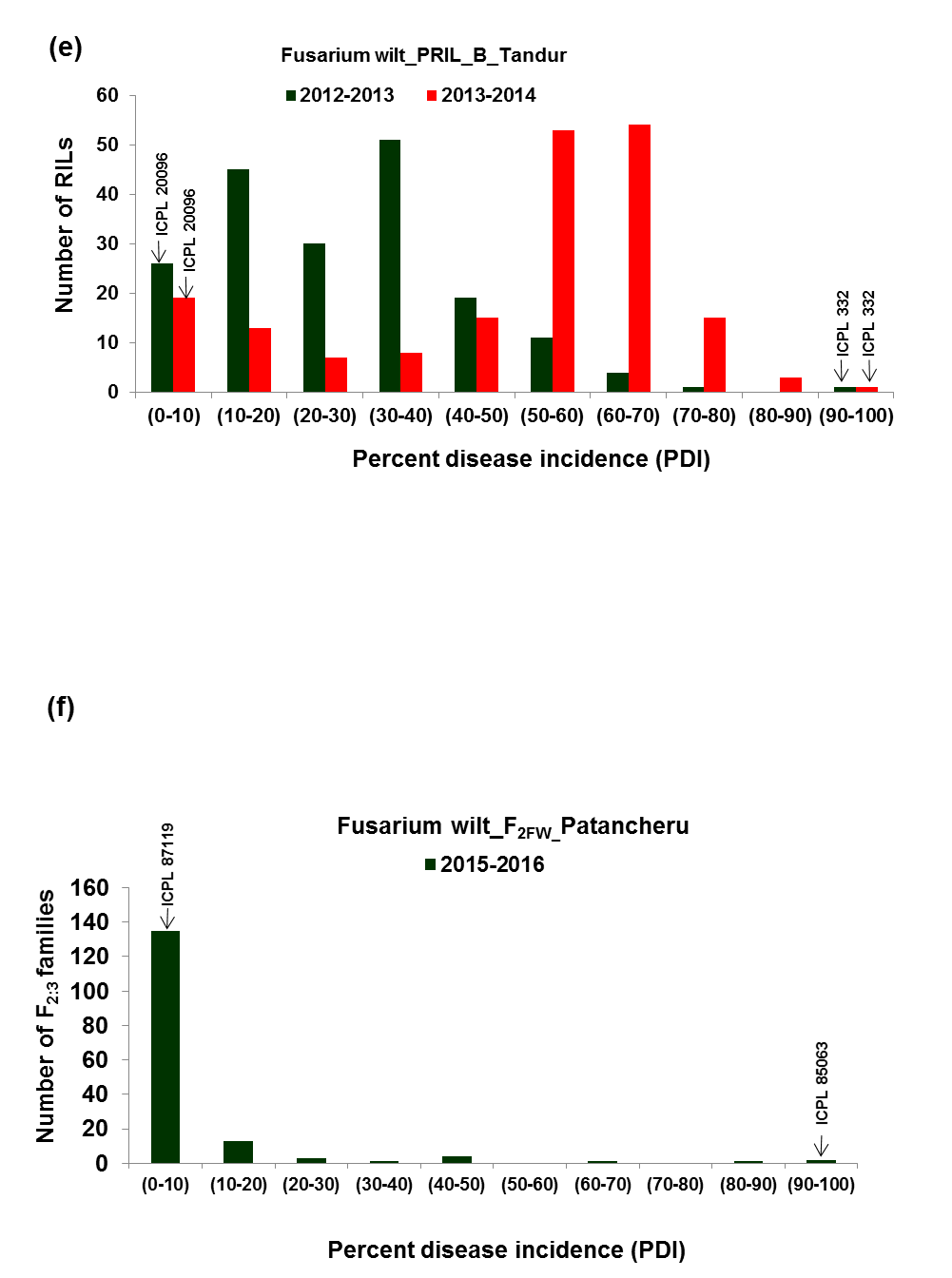


**Supplementary Fig. 1.**

Frequency distribution of percent disease incidence (PDI) for fusarium wilt (FW) resistance in various populations at different locations and years. The disease scoring was done on the basis of percentage of affected plants wherein 0 % means complete resistance while 100 % means complete susceptibility to FW. The PDI was monitored for two consecutive years (2012-13, 2013-14) in ICPL 99050 × ICPB 2049 (PRIL_A) and ICPL 20096 × ICPL 332 (PRIL_B) and population while for one year (2015-16) in ICP 85063 × ICPL 87119 (F_2:FW_) population. The PDI was divided into 10 categories and number of families in each category were calculated and plotted as bar plot. The PDI in ICPL 99050 × ICPB 2049 (PRIL_A) population at Patancheru location and Gulbarga location is shown in **a** and **b**. Figure **c**, **d** and **e** represent PDI in ICPL 20096 × ICP 332 (PRIL_B) population at Patancheru, Gubarga and Tandur, locations respectively, while, the figure **e** represents the PDI in ICP 8863 × ICPL 87119 (F_2_) population at Patancheru location.

**
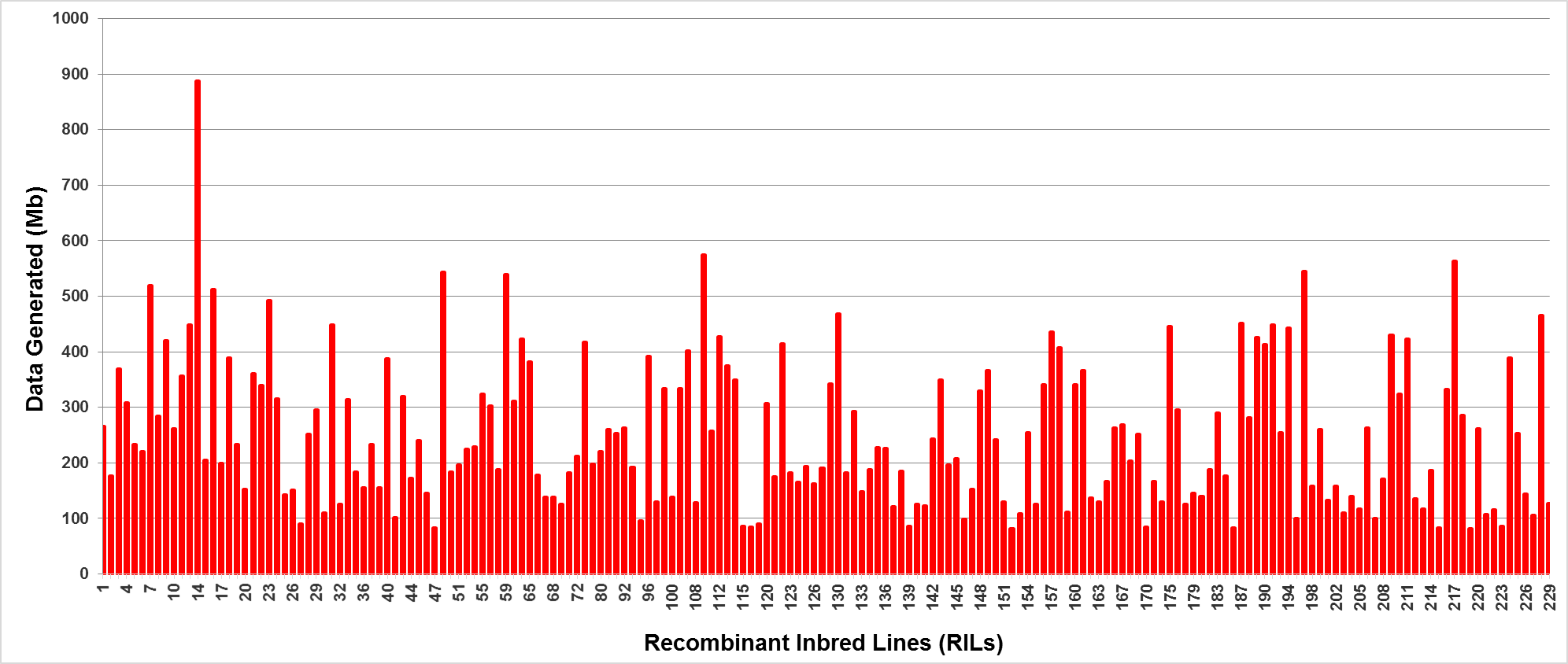
**

**Supplementary Fig. 2.** Data generated (in Mb) per sample in PRIL_A population derived from the cross ICPB 2049 × ICPL 99050

**
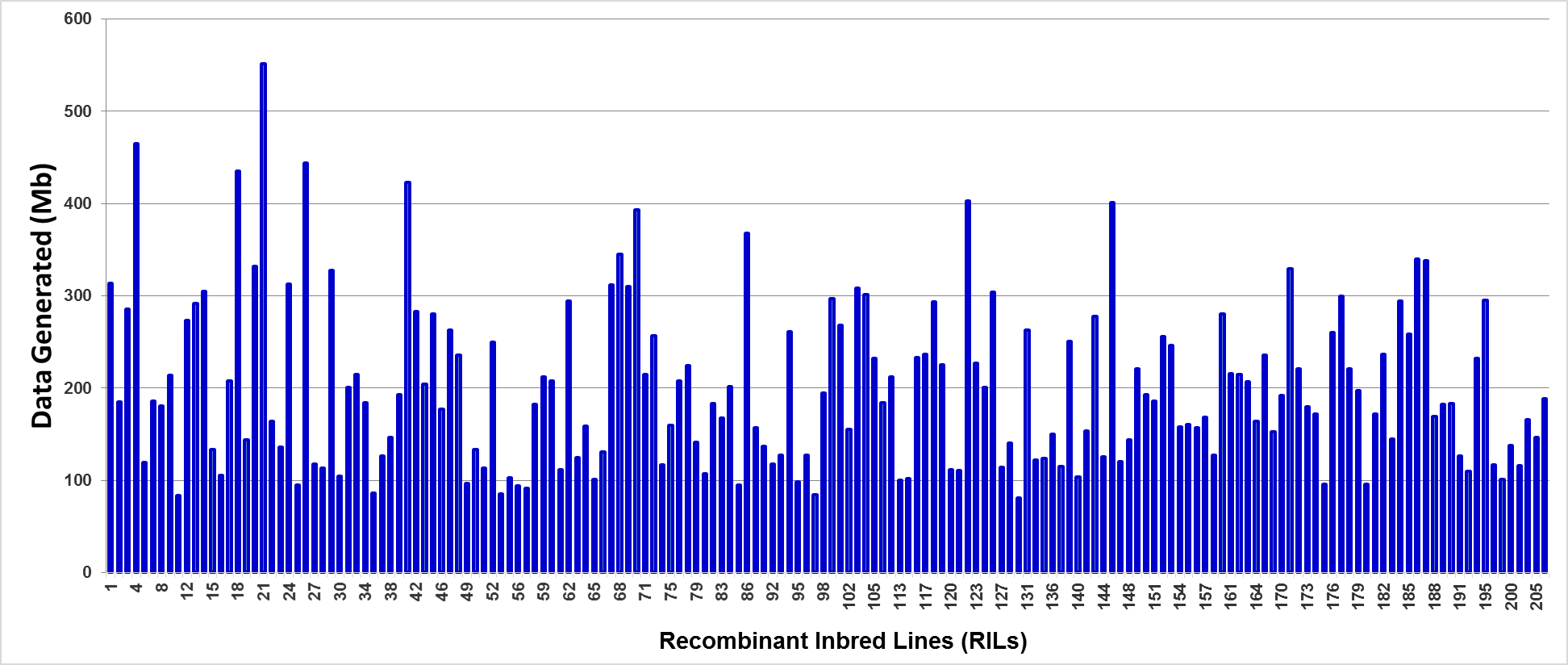
**

**Supplementary Fig. 3** Data generated (in Mb) per sample in PRIL_B population derived from the cross ICPL 20096 × ICPL 332

**
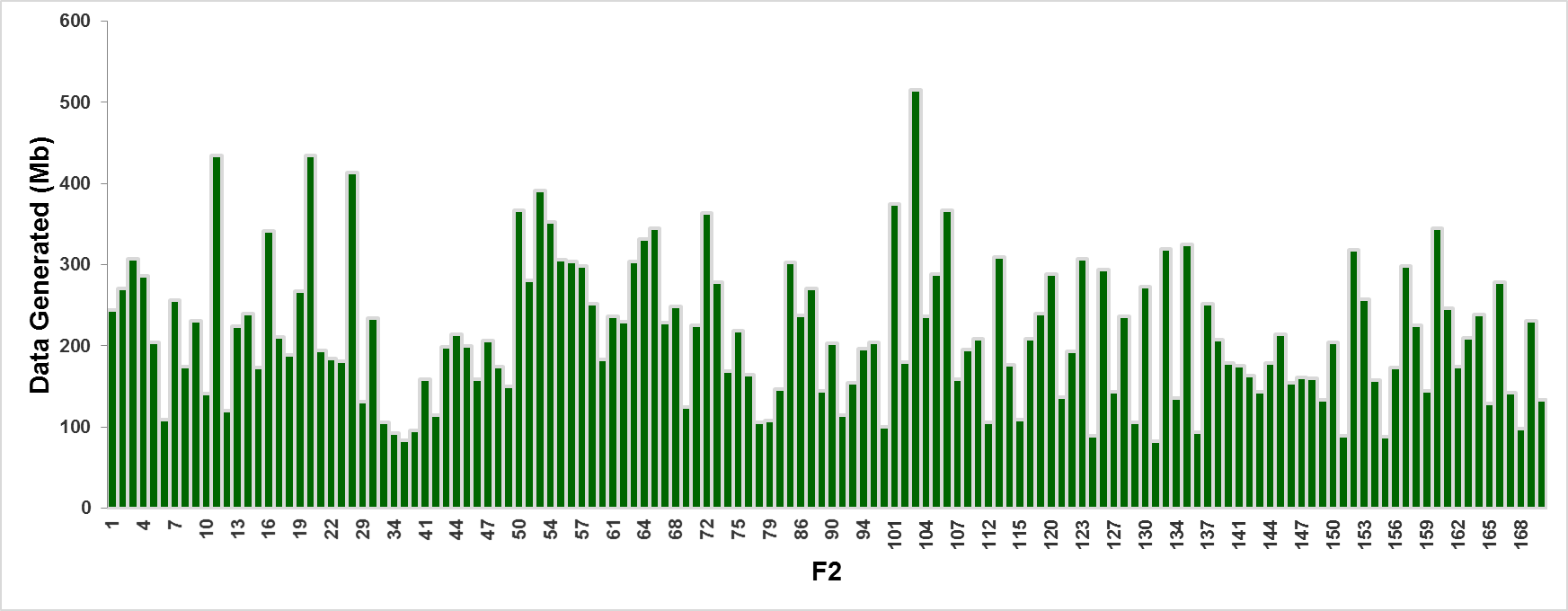
**

**Supplementary Fig. 4.** Data generated (in Mb) per sample in F_2_ population derived from the cross ICPL 85063 × ICPL 87119

**Supplementary Table S1** Main-effect QTLs (M-QTLs) for FW resistance identified in two RILs and one F_2_ population

| Trait | PRIL_A (ICPB 2049 × ICPL 99050) | | | | | PRIL_B (ICPL 20096 × ICPL 332) | | | | | F_2_ (ICPL 85063 × ICPL 87119) | | | | |
| --- | --- | --- | --- | --- | --- | --- | --- | --- | --- | --- | --- | --- | --- | --- | --- |
|  | No of QTLs | QTLs linkage group | Stable QTLs | Consistent QTLs | PVE (%) | No of QTLs | QTLs linkage group | Stable QTLs | Consistent QTLs | PVE (%) | No of QTLs | QTLs linkage group | Stable QTLs | Consistent QTLs | PVE (%) |
| *FW* | 8 | 6 | 2 | - | 6.55-14.67 | 6 | 3 | 2 | 1 | 7.92-15.26 | 5 | 3 | - | - | 2.75-56.45 |

Stable QTLs: appeared in more than one location; Consistent QTLs (appeared in more than one year); PVE: Phenotypic variation explained
